# Energy storage and catalytic behaviour of cmWave assisted BZT and flexible electrospun BZT fibers for energy harvesting applications

**DOI:** 10.1038/s41598-024-52705-0

**Published:** 2024-02-01

**Authors:** Avanish Babu Thirumalasetty, Siva Pamula, Thiyagarajan Krishnan, Vaishnavi Khade, Pathan Sharief, Siva Kumar Kota Venkata, Srinivas Adiraj, Madhuri Wuppulluri

**Affiliations:** 1 Department of Physics, School of Advanced Sciences, Vellore Institute of Technology, Vellore, Tamilnadu 632014 India; 2 School of Electrical Engineering, Vellore Institute of Technology, Vellore, India; 3 Department of nanotechnology, Deagu Gyeongbuk Institute of Science and Technology, Deagu, South Korea; 4 Ceramic Composite Materials Laboratory, Department of Physics, Sri Krishnadevaraya University, Anantapuram, Andhra Pradesh 515003 India; 5 Defence Metallurgical Research Laboratory Kanchanbagh, Hyderabad, India; 6 Ceramic Composites Laboratory, Centre for Functional Materials, Vellore Institute of Technology, Vellore, Tamilnadu 632014 India

**Keywords:** Energy harvesting, Energy storage, Renewable energy, Ferroelectrics and multiferroics, Electronic devices, Electrocatalysis, Electrochemistry

## Abstract

High-performance lead-free Barium Zirconium Titanate (BZT) based ceramics have emerged as a potential candidate for applications in energy storage, catalysis for electro chemical energy conversion and energy harvesting devices as presented in this work. In the present study hybrid microwave sintered BZT are studied for dielectric, ferroelectric and phase transition properties. BZT ceramic exhibits tetragonal structure as confirmed by the Retvield refinement studies. XPS studies confirms the elemental composition of BZT and presence of Zr. Polarization versus electric field hysteresis loops confirms the ferroelectric behaviour of BZT ceramic. Encouragingly, the BZT showed a moderate energy storage efficiency of 30.7 % and relatively good electro chemical energy conversion (HER). Excellent catalytic activity observed for BZT electrode in acid medium with low Tafel slope 77 mV dec-1. Furthermore, electrospun nanofibers made of PVDF-HFP and BZT are used to make flexible piezoelectric nano generators (PENGs). FTIR studies show that the 16 wt% BZT composite ink exhibits a higher electroactive beta phase. The optimized open-circuit voltage and short circuit current of the flexible PENG exhibits 7Vpp and 750 nA under an applied force of 3N. Thus, flexible and self-powered BZT PENGs are alternative source of energy due to its reliability, affordability and environmental-friendly nature.

## Introduction

Many researchers focus on sustainable green energy conversion and storage systems to reduce energy crises and global air pollution. Due to increasing energy demand and higher energy prices the world’s only concern is energy. In the next two decades, the global energy demand is anticipated to double. Because of this, it became necessary to develop numerous methods for the generation of energy and its storage. Piezoelectric materials are potential candidates for energy harvesting and storage applications^1^. Though Lead zirconium titanate (PZT) and other lead based piezoelectrics are commertially accepted, EU and other Western nations have baned the usage of lead in many commodities due to its toxicity. due to this resolution, biocompatible and ecologically friendly lead-free piezoelectric material are in demand due to lead toxicity. Lead free materials with low density can also have advantages in several electronic device applications such as transducers, sensors, energy conversion and storage etc. Various materials are currently under reconsideration as potential substitutes for PZT, which may be effective in specific situations^2^. So, the present research is focused on lead free materials and finding other sources of energy that are cleaner and last longer, like solar, wind, hydel, mechanical movements, magnetic fields and electrochemical etc. So, the present research is focused on finding other sources of energy that are cleaner and last longer, like solar, wind, hydel, mechanical movements, magnetic fields and electrochemical etc^3^. Therefore, renewable energy contributes to the evolution of various energy storage devices. This includes all energy storage devices like batteries, dielectric and electrochemical capacitors and electrolyzers^4^. The energy storage mechanism and flow of charges influence the power density, energy storage density and charge-discharge time of the devices. Batteries possess a high energy density but low power density^5^. The reason for this is the slow movement of charges. Consequently, they are utilized for long-term usage and low voltage applications (less than 5V)^6^. Electro chemical capacitors have moderate energy density with improved power density. They are utilized in low voltage applications (less than 3V). The dielectric bulk ceramic capacitor is an insulating material that can be placed between the two conducting parallel plates^7^ . The dielectric ceramic capacitor stores electrical energy from electrostatic displacement caused by the electric field. Dielectric capacitors possess low energy and high power densities, the charge and discharge capability of dielectric capacitors are faster than batteries (< 100 ns)^8^. Due to this reason the dielectric bulk ceramic capacitors are considered as energy storage devices for pulse power systems like electric vehicles, power grids etc^9^. Most importantly, dielectric ceramic capacitors have a superior mechanical and thermal stability compare to batteries (up to 250) and the inherent capacity of the dielectric bulk ceramic capacitors to store and deliver energy in rapid way, because it depends on polarization and depolarization in response to outside electrical fields instead of chemical reactions^10,11^. Which is essential for real-time promising energy storage applications in pulse power systems. Furthermore, various research efforts have been focused on investigating the electrochemical studies for the catalytic behaviour of BZT for green energy generation. Therefore, catalytic reduction technology (CRT) has garnered much interest due to its potential to generate green energy. Finding catalysts with excellent performance is one of the most critical tasks. Ferroelectric materials (FM) have recently been considered to be potential candidates for good- chemical reaction performance. This is because it is anticipated that ferroelectric materials will be able to overcome the limitations that are imposed by the Sabatier principle. The multiferroic $BiFeO_{3}$ can reduce the anode’s charge recombination rate from 17 to 0.6 $s^{-1}$ and the OER rate is also improved four times^12^. The spontaneous polarization of FM is primarily responsible for the enhancement. Ferroelectric materials have different electrostatic potentials and ways of distributing electrons, their surfaces will have different chemical activities and redox reactions. Therefore, ferroelectric switching can be used to modify the chemical characteristics of catalyst surfaces to increase catalytic efficiency. As a result, the use of ferroelectric materials in catalysts opens up new possibilities for highly effective new catalysts^13^. Additionally, various research efforts have been focused to investigate the mechanical energy conversion for driving smart electronics to explore inventive power sources. In 2006, Wang et al. developed the first nanogenerator using the piezoelectric properties of ZnO^14^. Flexible piezoelectric nanogenerators have gained a lot of attention in the last decade, because of their superior flexibility and energy harvesting ability from the ambient sources (mechanical energy into electric energy)^15,16^. Compared with rigid piezoelectric inorganic semiconductors and ceramics (e.g., $WS_{2}$, *ZnO*, *GaN*, *InN* and *PZT*, *PLZT*, *KBT*, *NKBT*, $BaTiO_3$ (*BT*) ), the piezoelectric polymer (*PVDF*) and its copolymer based flexible piezoelectric nanogenerators are easy to make, lightweight and stable at high electric fields, making them suitable for flexible and wearable applications^17–19^ . Pure polymer-based PENGs have inherently less piezoelectric coefficients than piezoelectric ceramics, which limits their applications. Recent research has led to the development of polymer ceramic composite piezoelectric nanogenerators, which aim to improve the piezoelectric coefficients and performance of flexible piezoelectric nanogenerators (PENGs) without compromising the device’s flexibility. *PVDF* is a versatile polymer with numerous properties such as piezoelectric, ferroelectric, and dielectric. It has numerous scientific and technological applications. It has five crystalline phases known as $\alpha$, $\beta$, $\gamma$,$\delta$. *PVDF* with higher $\beta$ phase is proven to exhibit better piezoelectric it can be produced by various processing methods such as aneling, melt casting, spin coating quenching, poling, stretching and adding additives such as MWCNT^20^ , nano clay^21^ , ionic salt^22^, RGO^23^, BT^24^ etc. would further improve the piezo nature of the polymer. Furthermore, *PVDF* co polymer plays the significant role on energy harvesting. High voltage coefficients ($g_{33}$) and piezoelectric coefficients ($d_{33}$) compare to pure *PVDF* are the desirable properties of the chosen co-polymer. The $PVDF- HFP$ satisfies all the above-mentioned properties. $PVDF- HFP$ blended copolymer is utilised in the present work. However, purity, stoichiometry, and grain size of the ceramic and synthesized method and sintering technique have a significant impact on the BZT performance. The conventional sintering of BZT ceramics, both calcination and final sintering require high temperatures (>1300 ^∘^C). The utilization of microwave sintering not only lowers the sintering temperature at which the material is processed, but it also results in improved material’s density and microstructure, and electrical properties^25^. Multiple reports exist on the microwave sintering of BT ceramics^26^ . However, there aren’t many reports about the effect of hybrid microwave sintering (HMS) of BZT ceramics on energy storage and energy conversion studies.To achieve excellent energy storage, electro chemical energy conversion and energy harvesting performance of BZT electro ceramic, the properties of the BZT electro ceramic have been optimized. In the present study, the properties of BZT, viz structural, dielectric, morphology, elemental analysis and applications such as energy storage (bulk electro ceramic), energy conversion and energy harvesting (PENG) are investigated.

## Results

### XRD

**Figure 1:**
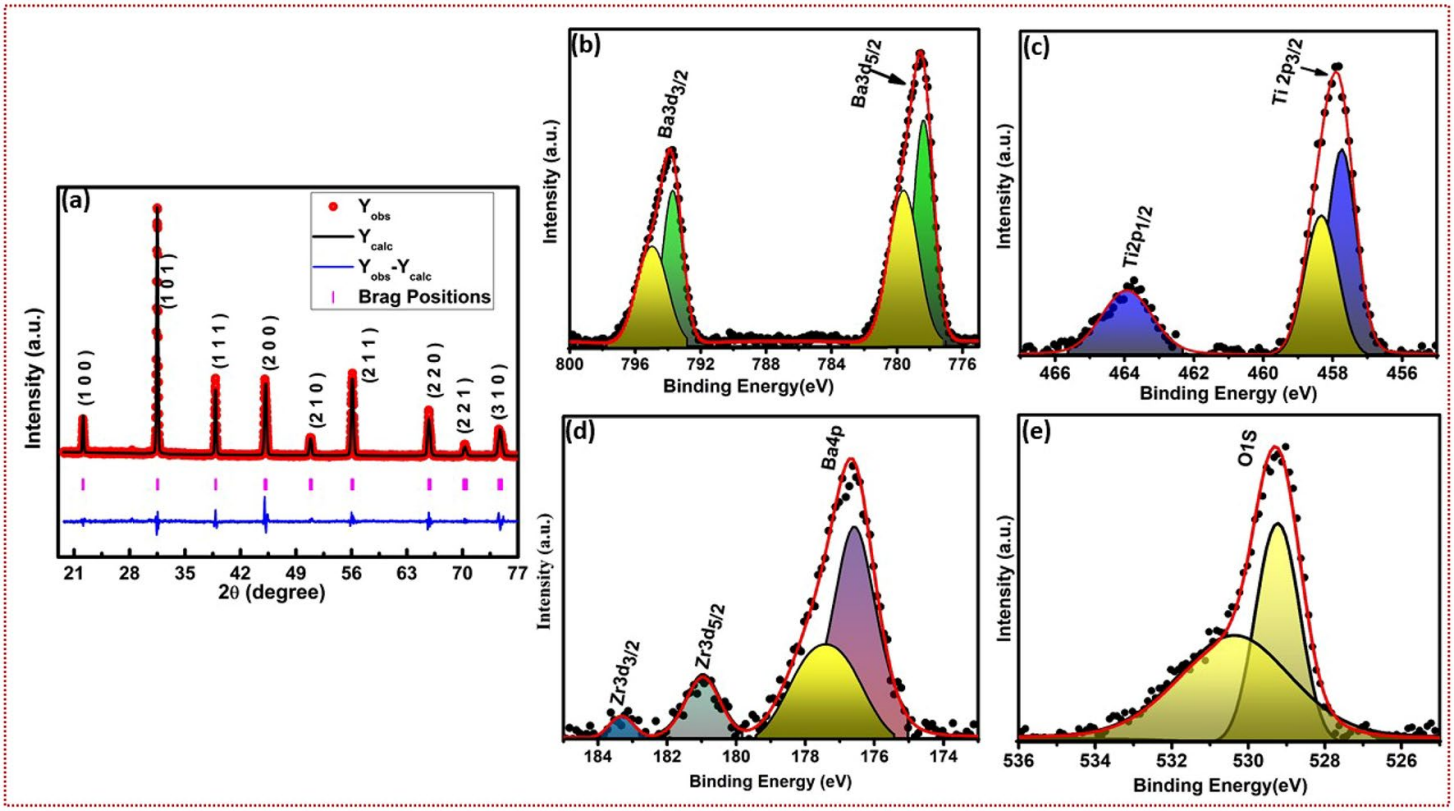
(**a**) Rietveld analysis for BZT indicates phase formation of BZT (**b**–**d**) An XPS image of the Ba-3d orbital states performed at a high resolution. Evidence of Zr can be found at the Ti-site in the Ti-2p state. The energies of the Zr-3d states contribute significantly to the range that spans from 180 to 184 eV. (**e**) High resolution scan close to the binding energy of Oxygen peaks. The peaks at 529.27 eV and 530.61 eV are attributed to $O^{2-}$, $O^{1-}$, and the chemically adsorbed oxygen ions.

The XRD profiles of hybrid microwave sintered ceramics (BT-1250, BZT-1250, BZT-1300) is shown in Fig. S1a (supplementary information). The XRD profile of hybrid microwave sintered BZT and BT sintered at 1300 and 1250 ^∘^C exhibit pure perovskite structure. One trivial impurity peak appears in the BZT XRD pattern near 2$\theta$ $\approx$26^∘^ indicate with (*) symbol , due to the low sintering temperature and is identified as $ZrO_{2}$ (JCPDS: 37-1484 and literature^27^) in Fig. S1a (supplementary information). The impure peak is attributed for non-uniform distribution of Zr ions. Among possible could be that the diffusion of Zr ions needs high temperature compare to Ti. No impure peaks are detected in the XRD profile of BT-1250 and BZT-1300, confirming that both the ceramics (BZT-1300 and BT- 1250) are of good quality with excellent crystallinity. The samples exhibit a tetragonal crystal structure with *P*4*mm* space group. The diffraction peaks are in good agreement with the literature as well as ICDD data base (ICDD- 00-005-0626)^28^ . The Rietveld analysis of BZT and BT is shown in Fig. 1a and S1b(supplementary information). The lattice constants of BT and BZT are a = 4.0116 Å, c =4.0150 Å and a = 4.0239 Å, c = 4.0110 Å respectively. The unit cell volume of BT and BZT are 64.6695 and 64.94 Å^3^ respectively. The goodness of fit ($\chi ^{2}$) value is 2.48 indicating a best fitting refinement of BZT. The average crystallite size of the BT and BZT are estimated by Scherrer formula given by the following equation^29^.

1 $$\begin{aligned} D=\frac{K\lambda }{\beta\, cos\theta } \end{aligned}$$

It is noticed that the estimated D value and unit cell volume of BZT is higher compare to BT. It may be due to addition of Zr to the BT and high sintering temperature of BZT compare to BT. The average crystallite size of the BT and BZT are 60, 91 nm respectively. The above values suggests diffusion of Zr ion in the unit cell. However, the above findings are further verified by XPS studies.

### XPS

To get a better understanding of the binding energies associated with the various chemical states of the material and presence of Zr in parent BT ($BaTiO_{3}$), X-ray photoelectron spectroscopy (XPS) is recorded. Fig. S2a,b (supplementary information) shows the survey spectrum of BT-1250 and BZT-1300 ceramics. The binding energies of BZT-1300 are tabulated in Table 1. Figure 1b–e depicts high-resolution spectra of BZT-1300 individual elements (Ba, Zr, Ti, O). The doublet spectra of Ba in BZT are Ba3d_3/2_, and Ba3d_5/2_ are fitted at higher energy 793.82 eV and lower energy 778.53 eV, respectively as shown in Fig. 1b. Similar binding energy peaks have been reported by Fahad et al.^30^ for the Ba3d_5/2_ and Ba3d_3/2_, where the former occurring at a lower energy of 778.34 eV and the latter occurring at a higher energy of 793.71 eV. This pattern of peaks is characteristic of the 2^+^ oxidation state of barium ions. Fig. 1c depicts high-resolution spectra of Ti. Due to the spin-orbit coupling, the high-resolution doublet spectra of Ti2p split into two peaks at 457.87 eV and 463.85 eV, corresponding to Ti2p_3/2_ and Ti2p_1/2_, respectively. The difference in binding energy between the major peaks of Ti2p in BZT is 5.98 eV, which is greater than the major binding energy peaks difference of Ti2p peaks of BT. The binding energy changes in Ti2p spectra in BZT ceramic is an evidence of Zr substitution at the Ti-site. The Zr substitution in the Ti site cannot create the reduction of Ti^4+^ to Ti^3+^^31^. Fig. 1d depicts high-resolution spectra of Zr. The high-resolution spectra of Zr3d_5/2_ and Zr3d_3/2_ are fitted at 180.94 eV and 183.34 eV, respectively. The difference in binding energy between the major peaks of Zr3d in BZT is 2.4 eV which is due to spin-orbit coupling the Zr ion substitution in the Ti site^30^. Fig. 1e depicts high-resolution spectra of O1s exhibit two overlapping peaks at 529.27 eV and 530.61 eV. Similar type of results were presented earlier by Chakrabarti et al.^32^.

**Table 1:** Comparison of the binding energy of elements between the present work and the literature.

S. no	State of electron		Binding energy	Binding energy
			(Present work)	(Literature)^30–32^
1	Ba3d	3d3/2	793.82	793.71
		3d5/2	778.53	778.34
2	Zr3d	3d3/2	183.34	183.5
		3d5/2	180.94	181.01
3	Ti2p	Ti2p1/2	463.85	463.74
		Ti2p3/2	457.87	457.95
4	O1s	O1s	528.98,530.91	529.5, 531

### Dielectric studies

**Figure 2:**
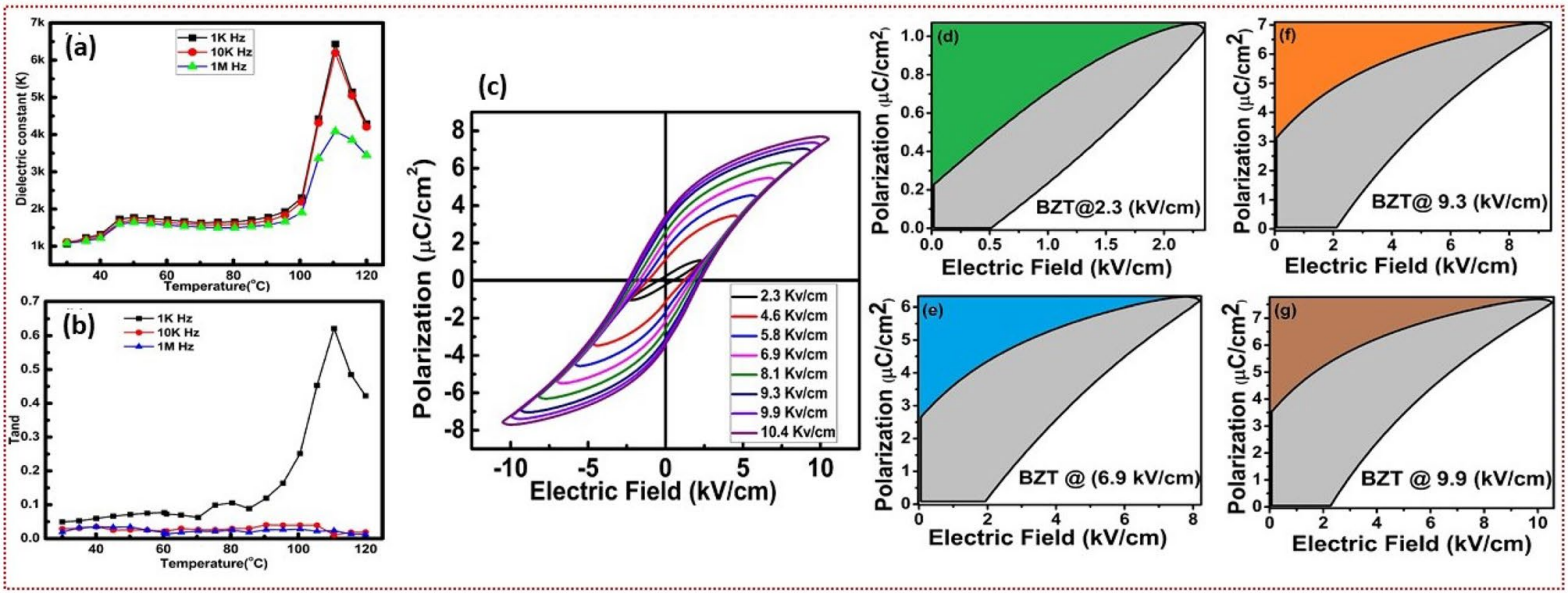
(**a**,**b**) Variation of dielectric constant and loss with varying temperatures at 1kHZ, 10kHz and 1MHz frequency follows the trend. The details are given in the text (**c**) P-E loops of BZT-1300 at various electric fields indicates the ferroelectric nature of BZT along with its capability to be utilized as energy storage device (**d**–**g**) energy storage properties for BZT-1300, the loop area gives the loss energy characteristics and the remaining area reveals the recoverable energy of the material.

**Figure 3:**
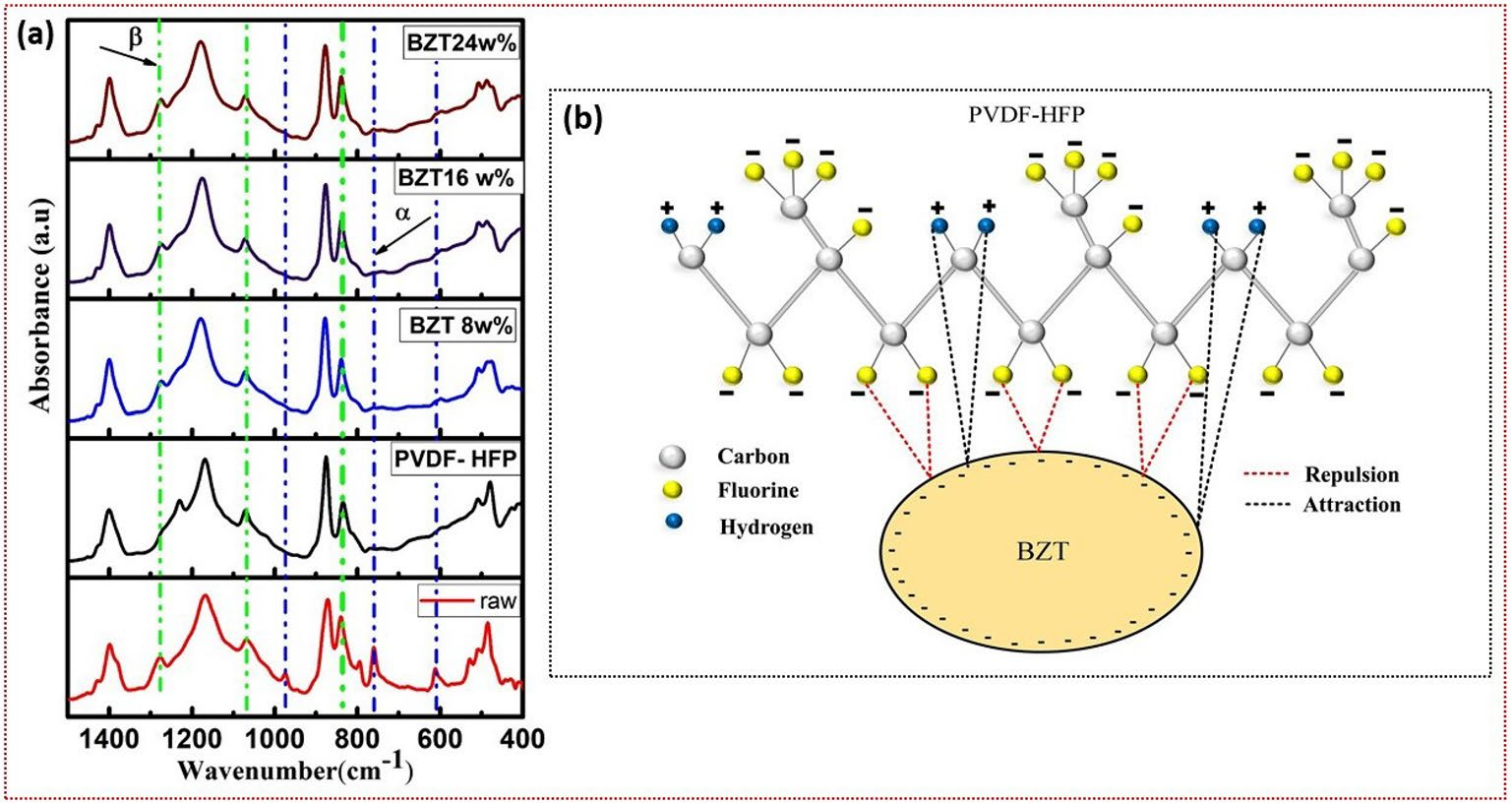
(**a**,**b**) FTIR spectra for $Ba Zr_{0.02} Ti_{0.98} O_{3}$ /PVDF-HFP composites with schematic illustrating zigzag TTT-conformation of the polymer chain because of interaction between the ceramic and polymer.

The temperature-dependent dielectric constant and loss behaviour of microwave-sintered BZT-1250, BZT-1275, BZT-1300 are investigated systematically in the range of temperature (30 to 120 ^∘^C), and frequency range of 1000 Hz to 1 MHz it is shown in Fig. S3a–d (supplementary information) and Fig. 2a,b. The relative permittivity and loss of BZT depends on several factors like purity, microstructure, chemical composition and sintering temperature etc^33,34^. It is noticed that the sintering temperature is directly proportional to dielectric constant and inversely proportional to T_c_ and Tan d. The reason may be the impure phase ($ZrO_2$) is presented at BZT- 1250, BZT- 1275. The dielectric results are well agree with structural studies. The dielectric constant and Tan d values are tabulated in Table 2. Figure 2a (BZT-1300) shows that up to 90 ^∘^C, the dielectric constant is almost the same at all frequencies. This is due to the weak and constant charge carrier feedback at these temperature ranges ( 30 to 90 ^∘^C)^35^. At room temperature to 110 ^∘^C the BZT shows tetragonal phase, which on further heating (greater than 110 ^∘^C) transforms into the cubic phase. The ferroelectric to para electric transition (tetragonal to cubic) of BZT-1250, BZT-1275, BZT-1300 are 118, 114. 108 respectively. It is shown in Fig. 3a,b. The T_c_ of BZT is lesser than BT the reason is the Substitution of Zr ion in the B site. Figure S4a,b (supplementary information) depicts the frequency-dependent relative permittivity and tand of BZT1300 which are in the range of 1 K to 1M. The microwave sintering temperature of BZT-1300 ^∘^C for 30 min appears to be optimal for producing high-quality BZT ceramic.

**Table 2:** Dielectric profile of BZT at different temperatures.

S. no	Material and sintering temperature	Dieelectric			Dielectric loss		
		1K	10K	1M	1K	10K	1M
1	BZT (1250)	2130	2100	1980	0.45	0.2	0.15
2	BZT (1275)	4150	4000	3580	0.38	0.22	0.1
3	BZT (1300)	6740	6500	4476	0.65	0.05	0.05

### Ferroelectric studies

P-E hysteresis loops are specific to ferroelectric materials and are used to verify the electro ceramics’ ferroelectricity. Figure 2c depicts the P-E loops of microwave sintered (1300) BZT ceramics at various electric fields. The P_r_ and E_c_ values can be found using ferroelectric hysteresis loops (P-E). The final polarisation values are almost never as high as predicted. Because the polarisation is dependent on the motion of the domain walls, the reorientation, and the switching between domains. The internal stress, as well as the internal bias electric field both, have an effect on the domain mechanism. The remanence polarization P_r_ of 3.47 $\mu$C/$cm^2$ and the lowest coercive field E_c_ of 2.26 kV/cm are recorded for the BZT-1300. Sharp edges of the PE loop of the present BZT indicate high resistivity, while round edges (lossy loop) indicate low resistivity. The square shape of the ferroelectric P-E hysteresis loop is characteristic of perfect ferroelectric material. Equation (2) can be used to determine the P-E hysteresis loop’s squareness (R_sq_). For an ideal case, the R_sq_ value of the PE loop is 2. In the present work, the microwave-sintered BZT (1300 ^∘^C) ceramics have an R_sq_ value of 0.58.

2 $$\begin{aligned} R_{sq}=\frac{P_{r}}{P_{s}} +\frac{P_{1.1Ec}}{P_{r}} \end{aligned}$$

Ferroelectric properties and energy storage properties of ferroelectric capacitors are determined by P-E hysteresis loops. The circum triangle formed by P_s_, P_r_ and the first quadrant curve represents the energy storage density (W_rec_). The first quadrant area under the curve represents the material energy loss density (W_loss_). From the P-E hysteresis loops the energy-loss density (W_loss_), recoverable energy-storage density (W_rec_), and the efficiency of the bulk ceramic capacitor are estimated from the following equations^9,36^.

3 $$\begin{aligned} W=\int _{0}^{Pmax} Edp \end{aligned}$$

4 $$\begin{aligned} W=\int _{P_r}^{Pmax} Edp \end{aligned}$$

5 $$\begin{aligned} \eta = \frac{W_{rec}}{(W_{rec}+W_{loss})}, \end{aligned}$$

where E is the applied electric field, P_max_ is the maximum polarization, P_r_ is the remnant polarization. The energy density values of the microwave-sintered BZT ceramics are found to be moderate energy storage. The energy storage efficiency of the BZT electro ceramic depends on loss and recoverable energy. The Polarization (P_max_), coercive field (E_c_) and remanent polarization (P_r_) values of perovskite BZT is directly proportional to the applied electric field. But the storage efficiency of the BZT is inversely proportional to the applied electric field. At low-applied electric fields, the storage efficiency ($\eta$) is high compared to higher fields. The recoverable energy and loss energy with the variation of the electric field of BZT is shown in the Fig. 2d–g. Despite high dielectric constant the observed low energy storage values are attributed to low squreness factor of the PE loops. The P_r_, P_max_, E_c_, W_loss_, W_rec_ and efficiency values of BZT with the variation of electric field are tabulated in Table S1. Table 3 shows the comparison of energy storage efficiency with literature.

**Table 3:** Comparison of output performance (energy storage efficency) of the BZT with the literature.

Composition	Sintering temperature (^∘^C)	Pr ($\mu$C/$cm^{2}$)	$P_{max}$ ($\mu$C/$cm^{2}$)	$\eta$ %	Ref.
$BaZr_{0.15}Ti_{0.85}O_{3}$	1270 (4 h)	19	34.5	50	^37^
BTSZ	1400 ( 2 h)	6.5	16	57	^38^
0.95 BT- 0.05 BZN	–	5.2	16.5	40	^39^
0.999BT- 0.001BY	1225 (2 h)	11.5	17.5	21.3	^40^
BCZT	1350–7 h	6	15	37	^41^
$BaTi_{0.9}Ce_{0.1}O_{3}$	1500–9 h	6	16.5	39	^42^
BZT	1300–30 min	3.5	7.6	30.7	Present work

### FTIR analysis

Figure 3a depicts the FTIR spectra of the BZT/PVDF-HFP composites. Bands at 763 $cm^{-1}$, 795 $cm^{-1}$, and 976 $cm^{-1}$ are characteristic of the non-polar $\alpha$ phase of the TGTG. The TTTT confirmation of the polar electroactive $\beta$ phase is responsible for the characteristic peaks at 841 $cm^{-1}$, 1276 $cm^{-1}$ , and 1431 $cm^{-1}$. In the meantime, the band that can be found at 840 $cm^{-1}$ is related to both the $\beta$ and $\gamma$ phases^28,43^ . The non-polar $\alpha$ phase do not appear at all in the spectra of electro spun samples. In spite of this, the 840, 1276, and 1431 $cm^{-1}$ bands, which correspond to the polar electro-active $\beta$ phase, are present in the spectra of all electro spun samples. The polar electro active phase ($\beta$ phase) relative fraction of electrospinning fibers are calculated by the following Eq. (6). In this scenario, it is assumed that the Lambert-Beer law governs the infrared transmittance.

6 $$\begin{aligned} F(\beta )= \frac{A_{\beta }}{(\frac{k_\beta }{k_\alpha })A_{\alpha }+A_{\beta }} \end{aligned}$$

where A$\beta$ and A$\alpha$ are the absorbances at 841 and 763 $cm^{-1}$, and K$\beta$ and K$\alpha$ are the absorption coefficients at those wavenumbers (6.1*104 and 7.7*104 $cm^{2}$ $mol^{-1}$, respectively). Figure 3a depicts the beta phase variation of composite fibers and pure polymer.

**Figure 4:**
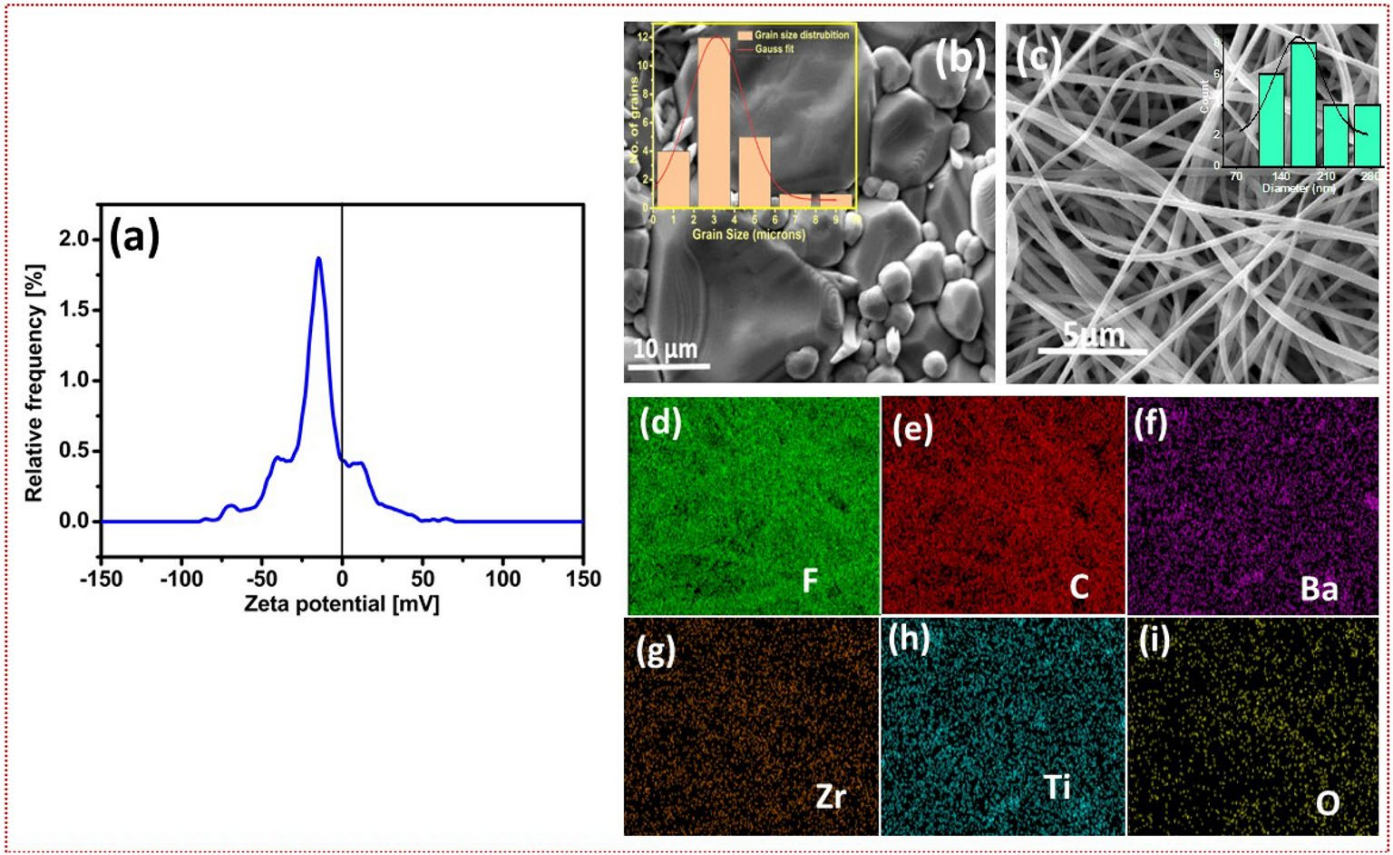
(**a**) Zeta potential analysis of BZT-1300 ceramics indicating the surface of BZT-1300 is negatively charged (**b**,**c**) SEM images for microwave sintered BZT with distorted grains with average grain size of 2.7 $\mu$m and electrospun interwoven BZT nanofibers with average diameter in micron range (**d**–**i**) Elemental mapping for electrospun BZT nanofibers.

The percentage of $\beta$ phase F($\beta$) estimated from Eq. (6), of BZT/PVDF-HFP composite film increases with BZT ceramic load compared to the pure polymer film. The polar phase value reaches up to $83\%$ when the content of BZT ceramic is 16 $wt\%$, which is higher than the value for pure PVDF and other BZT ceramic loadings. Further addition (24 wt%) of BZT ceramic reduces the F($\beta$) value of the BZT/PVDF-HFP composite film due to segregation, it can be shown in morphology of composite fibres (SEM high load BZT image). The beta phase percentage of the composites are tabulated in Table S2 (supplementary information). Figure 3b depicts a mechanism of interaction between polymer and ceramic that could account for the observed increase in the relative content of the polar $\beta$ phase. Also, zeta potential analysis resulted in a -Ve charge on the surface of BZT-1300 ceramic. The results are presented in Fig. 4a. Ceramic fillers with negative surface charges seems to increase the polar $\beta$ phase of polymers, a phenomenon attributed to the interaction between the negatively charged particles and the PVDF -$CH_{2}$ groups. Thus, the electrostatic attraction/repulsion between -$CH_{2}$/-and BZT (-Ve charge particles) and the $CF_{2}$ dipoles of polymer helps the PVDF-HFP chains to easily align in TTTT formation. Moreover, the attraction and repulsion forces play a significant role during the electrospinning process, which can enhance the polar $\beta$ phase. In the present work BZT plays the significant role forming $\beta$ phase.

### SEM analysis

The dense and tightly packed grains are formed in BZT sintered at 1300 ^∘^C (see Fig. 4b. The average grain size of the BZT is 2.7 $\mu$m as studied by the histogram images of ImageJ software. The water wave patterns noticed on the surface of the grains are suspected to be a ferroelectric domain layers. A deeper probing of the grain could confirm further details. Similar morphology was earlier reported by Fahad et al.^30^. The morphology of electro spun composites exhibited a high-quality smooth nanofiber without ceramic or polymer bead formation (Fig. 4c and Fig. S5a–c (supplementary information) except for 24 wt% ceramic loading. It is clearly seen that with an increase in BZT concentration, fibres showed a tendency to shrink in diameter. It may be due to change in viscosity of the solution and agglomeration of ceramic in the polymer matrix^44^. Similar types of results are published earlier by Athira et al.^28,45^. The distinct elemental mapping confirms all elements are uniformly distributed. It can be seen in Fig. 4d–i.

### Device results

**Figure 5:**
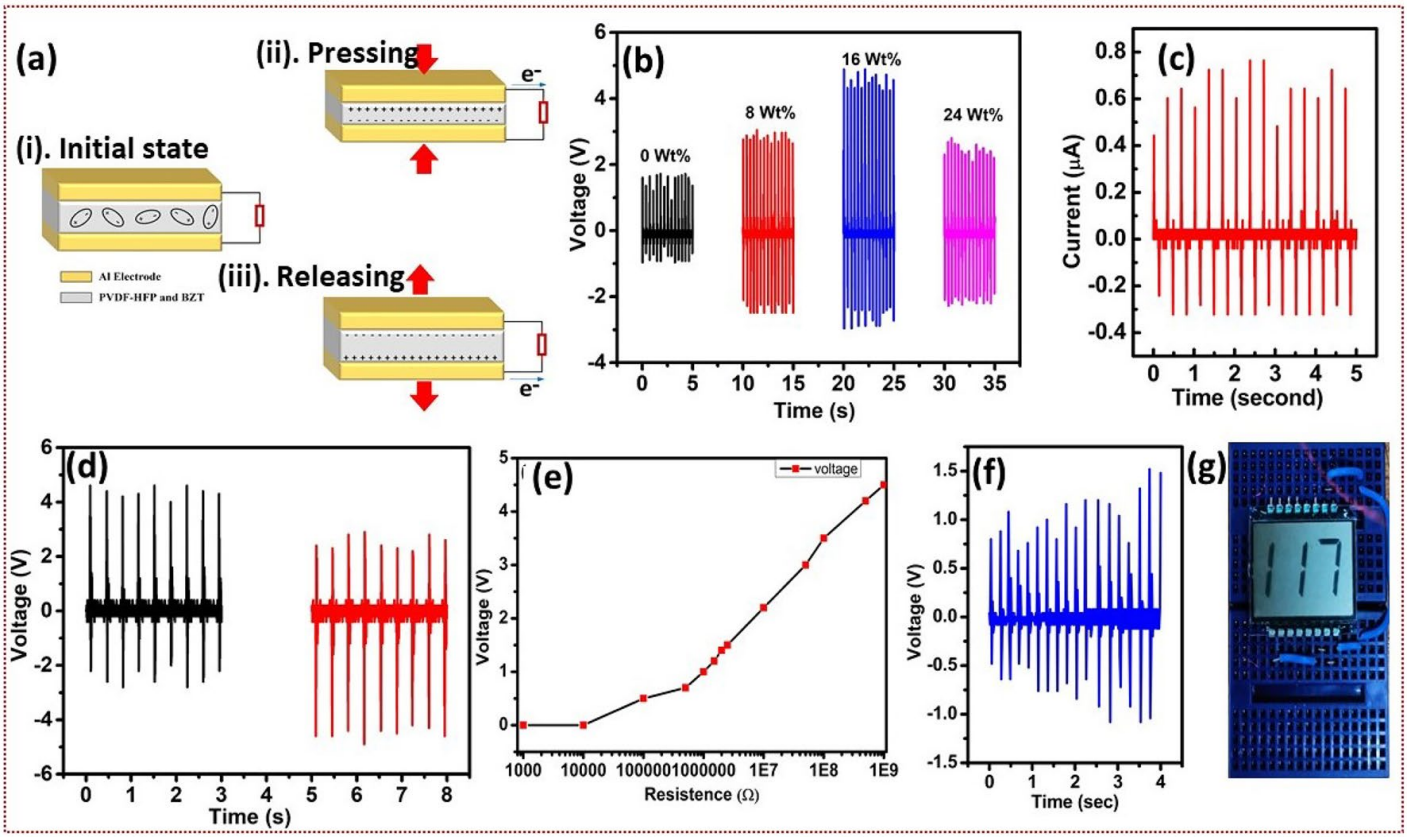
(**a**,**c**). (**a**) Schematic of PENG working mechanism. When an external force is applied to a crystal, it results in the displacement of anions and cations, leading to the creation of an electric dipole moment. This dipole moment can then generate a voltage differential in the direction of the applied tension within the material. The phenomenon described enables the conversion of mechanical force into electricity, and vice versa (**b**) Time dependent output voltage (Voc) of the PENG device at various wt% of BZT (0,8wt%, 16wt%, 24wt%) (**c**) Time dependent output current of the PENG device (**d**) Output voltage of reverse and forward connections of PENG (**e**) Effect of external load resistance on the output of the PENG (**f**) Finger tapping (**g**) Photographic image of LCD.

The focus of the work is to study the real time applications of the piezoelectric nano composites. To fabricate planner device, BZT composites are electro spun in to flexible fibers. The device is fabricated according to the schematic shown in Fig. 7. For mechanical strength, flexibility and to avoid interaction with the ambient atmosphere, the device is encapsulated with polyethylene terephthalate (PET). Further the encapsulation prevents slipping of the layers and ensures electrodes are intact during measurement. To characterise the device, the piezoelectric nanogenerator is placed on a vibration-free table to eliminate the possibility of a spurious voltage build up. The device was wrapped in aluminium tapes and grounded outside so electromagnetic waves wouldn’t mess with the data, and works as a Faraday cage^46,47^. The output performance of the flexible piezoelectric nanogenerator is investigated systematically under the constant cyclic force of 3 N at 3 Hz frequency using a linear mortar. When the BZT ceramic and PVDF- copolymer interact, charge density is created over the ceramic polymer (BZT- PVDF-HFP) composite, which causes surface charges to induce electrostatic interactions. The local stress results in spontaneous polarization of the piezo-electrically active beta-phase (Fig. 3b)^45,48^. The main causes of the polymer ceramic composite’s transition from the nonpolar phase to the electroactive beta-phase are the (–CH_2_/- CF_2_) bond stretching vibrations, the interaction of BZT ceramic dipoles with the polymer PVDF-HFP dipoles, and stress-induced polarization^49^. In this particular instance, the BZT ceramic plays the role of a nucleating agent for the formation of the polar crystalline phase within the PVDF-HFP/BZT ceramic composite^49^. Figure 5a depicts the schematic representation of the PVDF-HFP/BZT composite’s working mechanism. If there is no mechanical impact from the outside, all dipoles in the composite (PENG) are randomly oriented, so there is no net dipole moment, as shown in Fig. 5(i). The crystal structure of the flexible piezoelectric nanogenerator PENG is deformed under stress, changes the dipolar distribution and results in potential across. A piezoelectric potential is created by the difference in potential between the two electrodes, which propels the flow of electrons through an external circuit from one electrode to another electrode. It leads to the + ve voltage. It is shown in Fig. 5(ii). The piezoelectric potential vanishes in a rapid way when the strain is released, and to make up for this, the electrons that have accumulated close to the electrode travel through the external circuit and back to the other electrode, which results in an electric signal travelling in the opposite direction leading to -ve potential (shown in Fig. 5(iii)). As a result, an alternating voltage is generated by the persistent vertical compression and release. The optimized output voltage of piezoelectric nanogenerators made from BZT (0, 8, 16, 24 wt%) under constant cyclic force of 3 N at 3 Hz frequency are presented in Fig. 5b. The output voltage is found to systematically increases with BZT load wt% of up to 16 wt%. Beyond which a declain in the output voltage is noticed which is as expected from the FTIR results. Fig. 5c depicts the output current of 16 wt% loaded BZT device. The two terminals of the PENG are connected to the oscilloscope to determine the energy harvesting from daily mechanical stress. The forward and backward switching tests were performed to find out whether the PENG’s output signals originate from the piezoelectric effect or not. Fig. 5d depicts the forward and reverse bias test results of the PENG; it is clear that when the device is connected in the reverse configuration, the output signals of the forward configuration are precisely switched exhibiting high efficiency of switching and performance quality. The voltage values of PENG increases as the load resistance is increased as shown in Fig. 5e. Figure S6 (supplementary information) shows the power across various resistance. Further, Fig. 5f shows the output performance of PENG under the finger-tapping test. The output voltage is good enough to activate LCD screen. This result is depicted in Fig. 5g and supporting video 1. Figure S7 (supplementary information) shows the stability(voltage response) of PENG. Table 4 shows the comparison of PENG (BZT-PVDF-HFP) output voltage with literature

**Table 4:** Comparison of the synthesis technique and output performance of the PENG with the literature.

Material synthesis technique	Output voltage (V)	Device dimensions	Thickness	Applied force	Ref.
PVDF- BT-Electrospinning	0.006	12 $cm^{2}$	20 $\mu$m	1 N	^50^
Zno/PDMS-Spin coating	1.6	15 $\times$ 15 $mm^{2}$	50 $\mu$m	2 N	^51^
BCZT nanowires	3.25	–	–	2 N	^52^
BT/CNT/PDMS-Tape casting	4.6	–	0.25 cm	–	^53^
PVDF- BT- Electrospinning	6	–	–	32 N	^54^
BT-PVDF-TrFE-3D- Printing	6	2 $\times$ 2$cm^{2}$	40 $\mu$m	60 N	^55^
GO-PVDF-Electrospinning	7	18 $\times$ 44 $mm^{2}$	0.8 mm	0.4 N	^56^
BZT/PVDF solution casting	8.64	–	–	500 N	^57^
BZT-BCT/PVDF- solution casting	9.75	–	–	500 N	^57^
BZT/PLA/PDA- solution casting	14.4	–	–	1.8 N	^58^
BZT- PVDF-HFP-Electrospinning	$7V_{pp}$	2 $\times$ 4 $cm^{2}$	30 $\mu$m	3 N	Present work

### Electrochemistry

**Figure 6:**
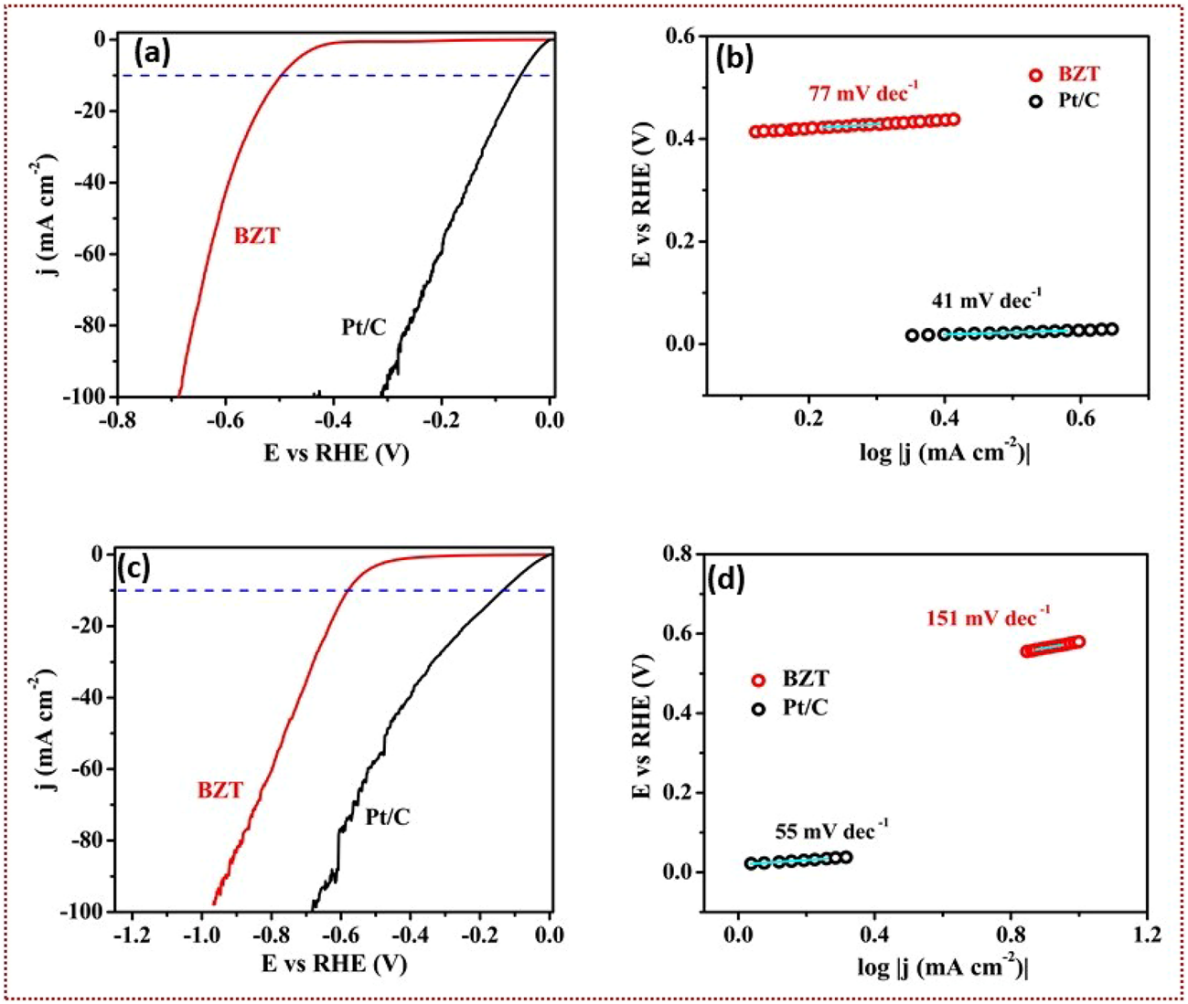
(**a**,**c**) HER polarization curves and Tafel plots of BZT in 0.5 M $H_{2}SO_{4}$ (**a**,**b**) and 1M KOH (**c**,**d**) electrolytes.

**Figure 7:**
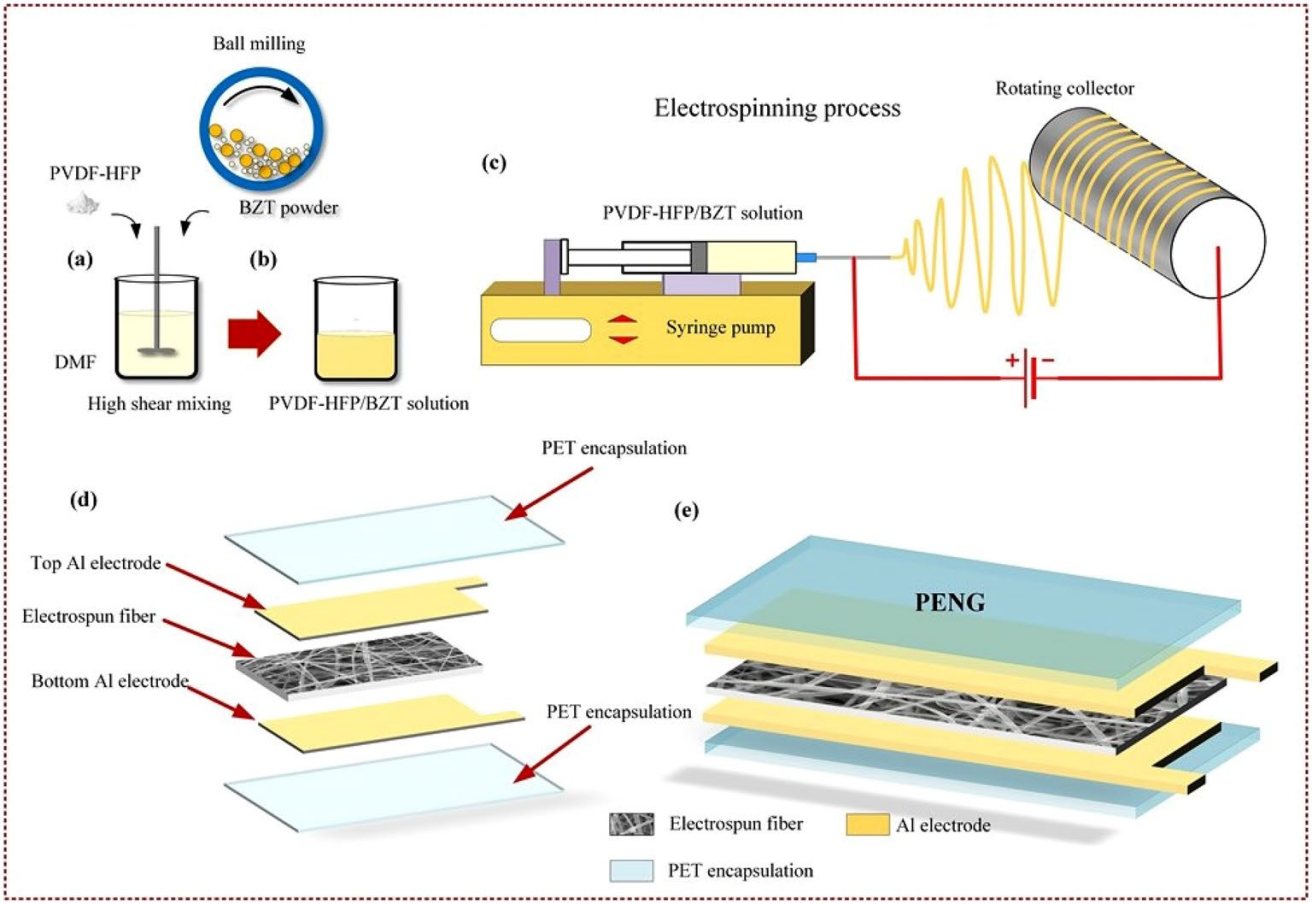
Schematic representation for (**a**–**c**) preparing BZT nanofibers using electrospinning which gives a high pace output at low cost. Producing micron-sized yarns that are made of nanofibers at a rate of 70 meters per minute allows for the formation of a variety of different assemblies (**d**–**e**) PENG device fabrication involving electrode installation and PET encapsulation steps.

Another interesting application of BZT ceramic is its capability to promote hydrogen evolution reaction (HER). Figure S8 (supplementary information) shows the schematic of water splitting. The HER performance of as prepared BZT is tested in the $N_{2}$ saturated 0.5M $H_{2}SO_{4}$ and 1M KOH electrolytes. Figure 6a–d shows the HER performance and corresponding Tafel plots of BZT measured in 0.5M $H_{2}SO_{4}$ and 1M KOH electrolytes with a scan rate of 20 $mVs^{-1}$. The observed over potentials are compared with commercially obtained Pt/C. From the plots it is evident that BZT is exhibiting higher overpotentials than the standard Pt/C electrode. BZT exhibited an overpotential of 490 mV (Fig. 6a- acidic medium) and 574 mV (Fig. 6c- alkaline medium) at standard 10 mA $cm^{-2}$, while Pt/C exhibited 42 mV (Fig. 7a-acidic medium) and 102 mV (Fig. 6c-alkaline medium) at standard 10 mA $cm^{-2}$. The obtained overpotentials for hybrid microwave sintered BZT are extremely superior to previous reports^59–63^. Table S3 shows the Tafel slope values comparison with literature. The electrocatalytic activity is plausibly explained based on Tafel slopes. Figure 6b,d exhibits the Tafel slopes for the microwave sintered BZT and commercially obtained Pt/C working electrode. The results once again confirm the superior nature of BZT as a electrochemical catalyst. The Tafel slope of BZT in the acid medium is 77 mV $dec^{-1}$ as compared to 41 mV $dec^{-1}$ of Pt/C. Though the variation in alkaline medium is very less, i.e., it is 166 mV $dec^{-1}$ for BZT and 152 mV $dec^{-1}$ for Pt/C electrode. BZT is found to be rapidly reactive in acidic medium than in alkaline medium. The observed small Tafel slope of 77 mV $dec^{-1}$ is attributed to rapid electron transport through the interfacial planes of BZT. Further, it is noteworthy that the slope of 77 mV $dec^{-1}$ is the lowest of the ferroelectric ceramics reported till date. The hydrogen evolution reaction (HER) mechanism can be analyzed based on Volmer (> 120 mV $dec^{-1}$), Volmer -Herovsky (> 120-40 mV $dec^{-1}$) and Volmer-Tafel (>40 mV $dec^{-1}$) kinetics^64–67^ . This reaction kinetic resembles the probable H- atom adsorption and desorption mechanisms to enrich the HER activity of the prepared catalyst. From the obtained Tafel slopes Pt/C electrode follows Volmer Tafel kinetics while BZT follows Volmer Herovsky. The sample is tested for CV cycles and is followed to be retracing the cycle for 2000 cycles. The stability of the catalyst is estimated by the polarization curves (LS - linear sweep voltammetry) before and after the 2000 CV cycles. The LSV curves coexists with after 2000 cycles of CV. This test confirms the stability of BZT as a catalyst. The results are presented in the supplementary (Fig. S9, Supporting Information).

## Discussion

In summary this manuscript reports the microwave sintering of BZT and its applications. The XRD refinement studies confirm the tetragonal crystal structure with the goodness of fit 2.48. The dielectric constant is found to be 6740 with a low dielectric loss of 0.65 at a frequency of 1KHz. The PE loop shows the moderate energy storage efficiency of 30.7 %. In order to show that BZT have a rapid reaction mechanism and excellent catalytic activity, electro chemical studies are performed. A Tafel slope of 77 mV dec^-1^ is observed for the acid medium. Furthermore, electrospun PVDF-HFP-BZT mats were used to make a flexible PENG device. Electrospinning effectively resulted the composite fibres with higher percentage of beta phase of PVDF-HFP - BZT. The piezoelectric outputs of the PENGs are greatly improved by the hybridization of negative surface charge on BZT ceramic particles and positively charge hydrogen in PVDF -HFP polymer. The open-circuit voltage, short circuit current and power of the nanocomposite fibre PENG with 16 wt% BZT exhibited 7Vpp, 750 nA and 2.5 $\mu$W under the mechanical force of 3 N. Furthermore, the voltages are developed by finger tapping   2 Vpp and LCD display could be switched ‘ON’. The feasibility studies present energy storage, energy conversion, and energy harvesting applications presenting great potential of the environmentally friendly BZT-based materials to be integrated in a wide range of applications.

## Methods

Barium zirconium titanate (BZT) ($Ba Zr_{0.02} Ti_{0.98} O_{3}$) and BT are synthesized by using regular solid-state double sintering method. To synthesize the BZT ferroelectric phase, the compositional powder materials, $BaCo_3$ (Aldrich 99.99%), $TiO_2$ (Aldrich 99.99%), $ZrO_2$(Aldrich 99.99%) are weighed in a suitable stoichiometric ratio and milled in a zirconium bowl using zirconium balls and acetone as grinding media for 8 hrs at 400 rpm in the high-energy planetary ball mill (PULUERISETTE-6). After milling, the slurry is dried at 60 ^∘^C for 2 hrs. The obtained powder is compacted by cold pressing and calcinated at 1000 ^∘^C (BZT and BT) for 20 min in an alumina crucible using a hybrid microwave furnace (VB Ceramics Pvt. Ltd. India). After that, the calcinated powders were milled at 8 hrs in an acetone medium. The slurry is dried and seaved using 10- micron mesh to get uniform powders. These homogeneous powder is added with a small drop of PVA for the pellet preparation. The set of green pellets (10 mm dia and 1mm thickness) are sintered in hybrid microwave furnace at 1300 ^∘^C (BZT), 1250 ^∘^C (BT) for 30 min in an open-air atmosphere.

### Electrospinning of BaZrTiO_3_/PVDF-HFP Nanofibers

To make the electrospinning solution, a mixture of DMF and acetone was used as a solvent. At first, PVDF-HFP is mixed into the solvent at a rate of 500 rpm for 2 hours at a temperature of 60 ^∘^C. The next step involves adding BZT powder to the transparent PVDF-HFP solution see in Fig. 7a,b. The solution is sonicated for 1 h to make a homogeneous solution. The ceramic weight percentage in the composite (BZT/ PVDF-HFP) series is set to 0, 8, 16, and 24 wt%. Initially, the metal needle is fixed at the end of the 10 ml plastic syringe. The homogenous formulated composite ink is filled with the syringe. Parameters for electrospinning include a 20 kV applied voltage, a drum speed of 1500 rpm, and a 1.25 ml/hr flow rate. Typically, there is about 15 cm gap is maintained between the drum and the needle. The fibres produced by electrospinning are collected on a collector. The collector is wrapped with the Al foil. See in Fig. 7c. Before characterization, the collected fibres are dried in vacuum at room temperature. The BZT/ PVDF-HFP series has ceramic weight percentages of 0, 8, 16 and 24 wt%.

### Device fabrication

Figure 7d–e shows the schematic of a PENG device with BZT/PVDF-HFP composite film. PENG is fabricated by slicing the electro spun flexible film to the required sizes. Two flexible Al sheets attached to both sides of the film will act as electrodes. To measure the output performance of the flexible device (PENG), copper wires are pasted on Al electrodes using conductive epoxy. The flexible adhesive PET sheet is fixed on both sides of the devices. This will eliminate the gaps between the electrode and PET sheet to avoid the triboelectric effect and protect the device from the surrounding atmosphere.

### Supplementary Information

Supplementary Information.

Supplementary Video 1.

## Data Availability

The corresponding author can provide the datasets used and/or analyzed in the current work upon reasonable request.
