# Supplementary material for: Energy storage and catalytic behaviour of cmWave assisted BZT and flexible electrospun BZT fibers for energy harvesting applications
Source: Sci Rep. 2024 Feb 1;14:2650. doi: 10.1038/s41598-024-52705-0 (PMC10834441; doi:10.1038/s41598-024-52705-0)
Supplement: Supplementary file 1 — Supplementary Information. [file 41598_2024_52705_MOESM1_ESM.docx]

**Energy storage and catalytic behaviour of cmWave BZT and flexible electrospun BZT fibers for Energy harvesting applications**

**Avanish Babu Thirumalasetty^a^, Pamula Siva^b^, Thiyagarajan Krishnan^c^, Vaishnavi Khade^d^, Pathan Sharief^e^, Siva Kumar Kota Venkata^f^, Srinivas Adiraj^g^, Madhuri Wuppulluri*^h^**

a, b, d, *Department of physics, School of Advanced Sciences, Vellore Institute of Technology, Vellore, 632014, Tamilnadu, India.; E-mail: a-* [*avani.thirumalasetty@gmail.com, b-*](mailto:avani.thirumalasetty@gmail.com,%20b-) [pamulasiva94@gmail.com](mailto:pamulasiva94@gmail.com), d- khade_vaishnavi@rediffmail.com

c *School of Electrical Engineering Vellore Institute of Technology Vellore, India;* [*c-thiyagarajreddy@gmail.com*](mailto:c-thiyagarajreddy@gmail.com)

e *Department of* Dept. of nanotechnology, Deagu Gyeongbuk Institute of Science and Technology, South Korea.: *E-mail:* *shareefpathan2012@gmail.com*

f *Ceramic Composite Materials Laboratory, Department of Physics, Sri Krishnadevaraya University, Anantapuram, Andhra Pradesh, 515003, India; sivakumar.sivani@gmail.com*

g *Defence Metallurgical Research Laboratory Kanchanbagh, Hyderabad, India; E-mail: adirajs.dmrl@gov.in*

∗h *Ceramic Composites Laboratory, Centre for Functional Materials, SAS, VIT, Vellore- 632014, Tamilnadu, India.; E-mail: madhuriw12@gmail.com*


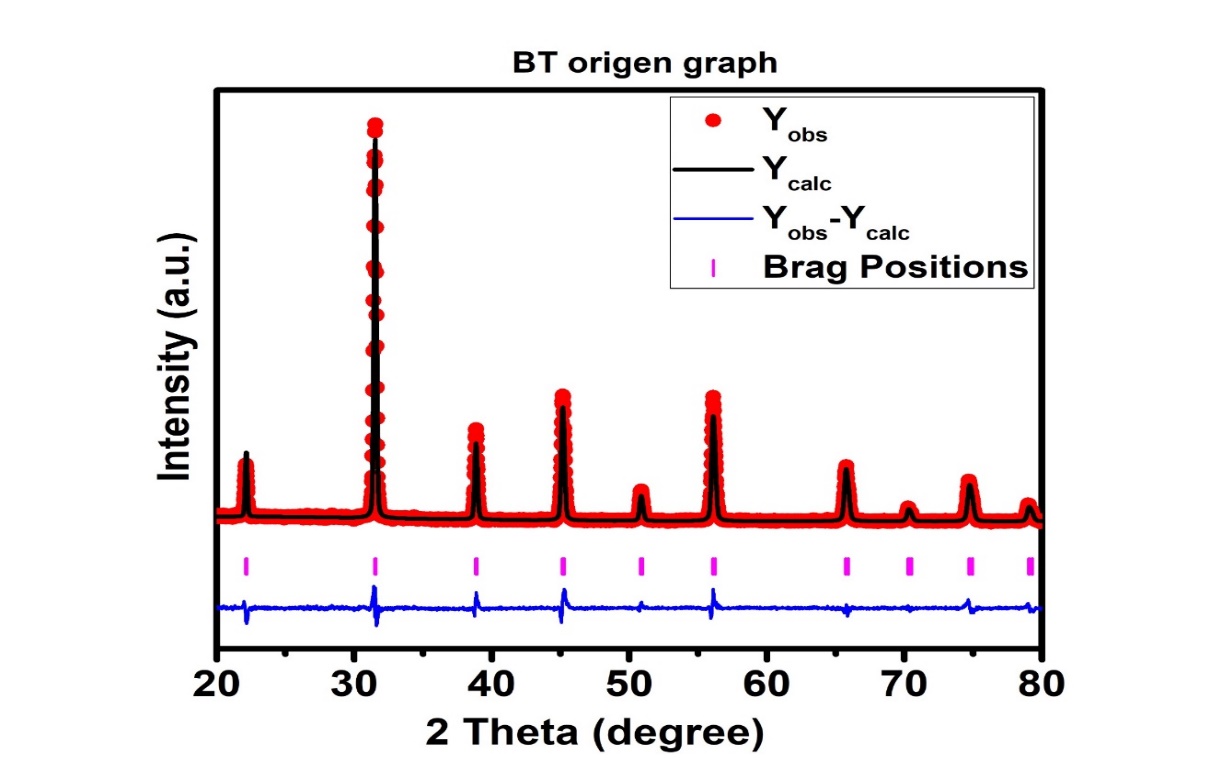

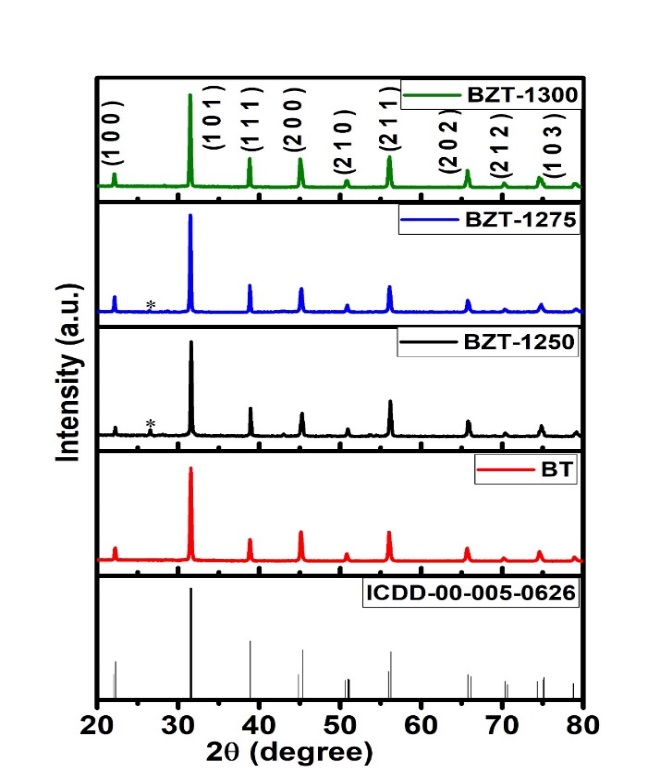


**(b)**

**(a)**

**Figure S1**: (a) XRD pattern of (BT, BZT-1250, BZT-1275, BZT-1300), (b) Rietveld refinement of BT-1250

**(a)**


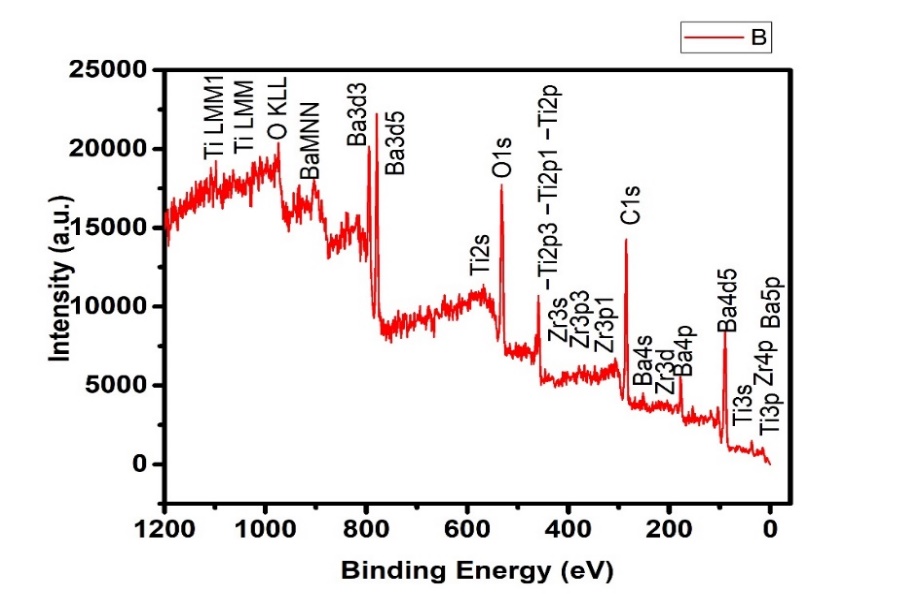


**BZT**


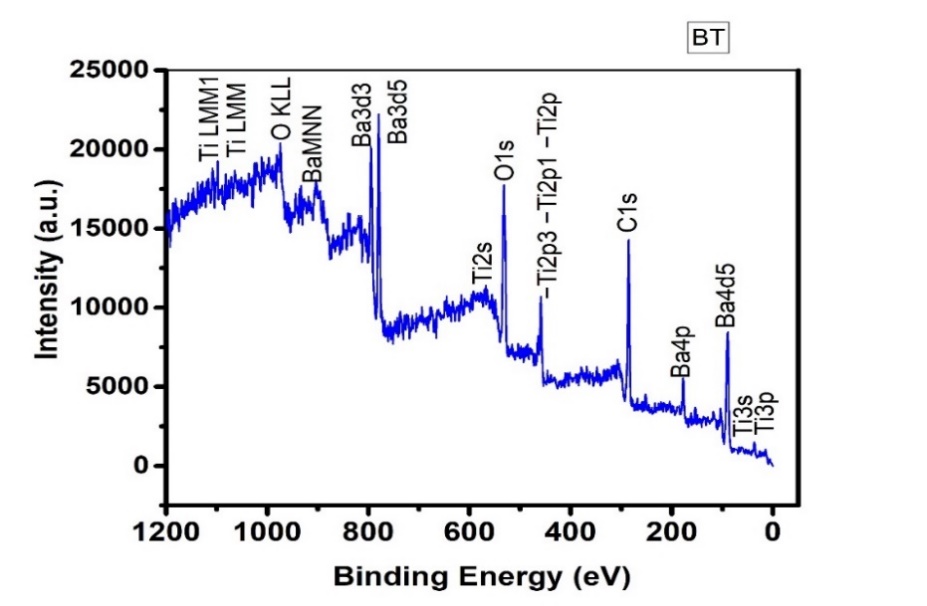


**BT**

**(b)**

**Figure S2:** (a) Survey spectrum of BT- 1250, (b) Survey spectrum of BZT-1300.


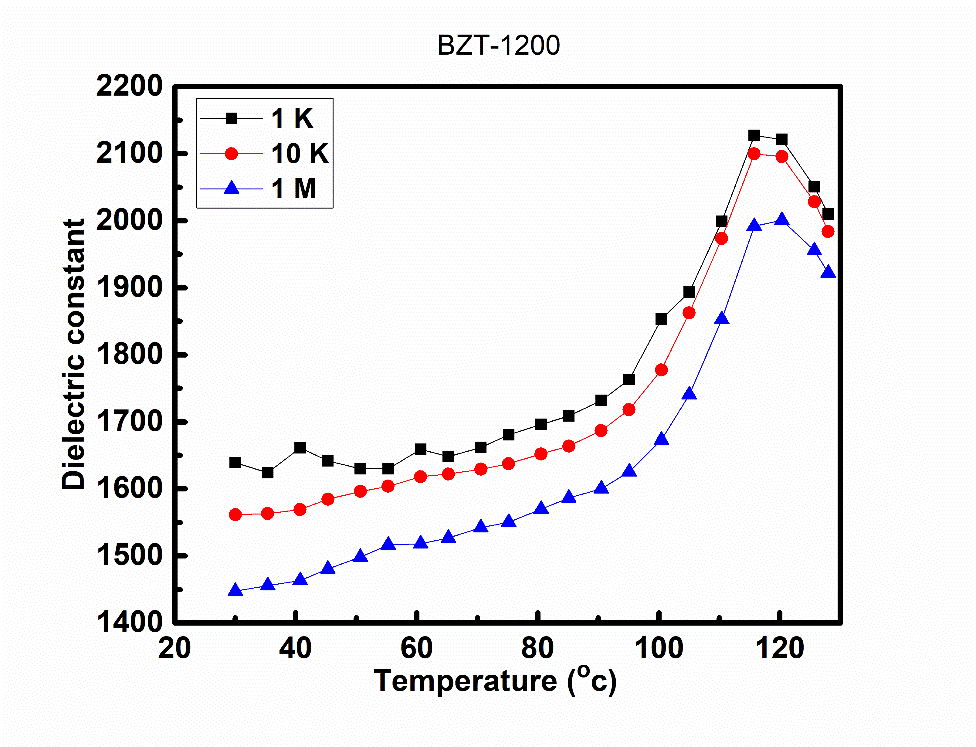

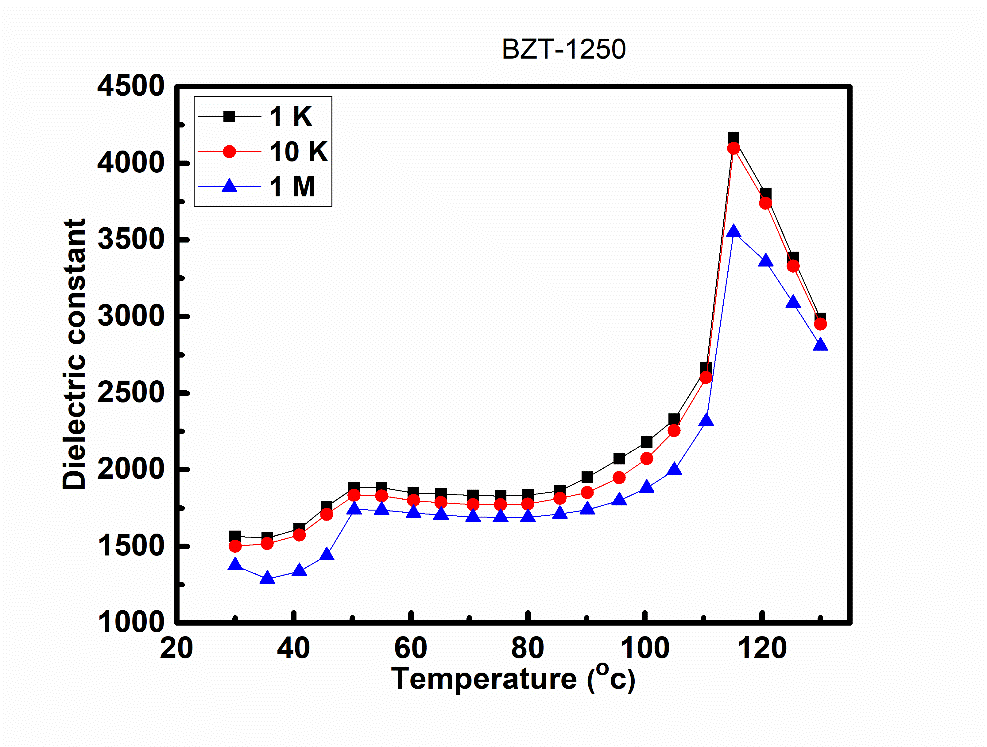

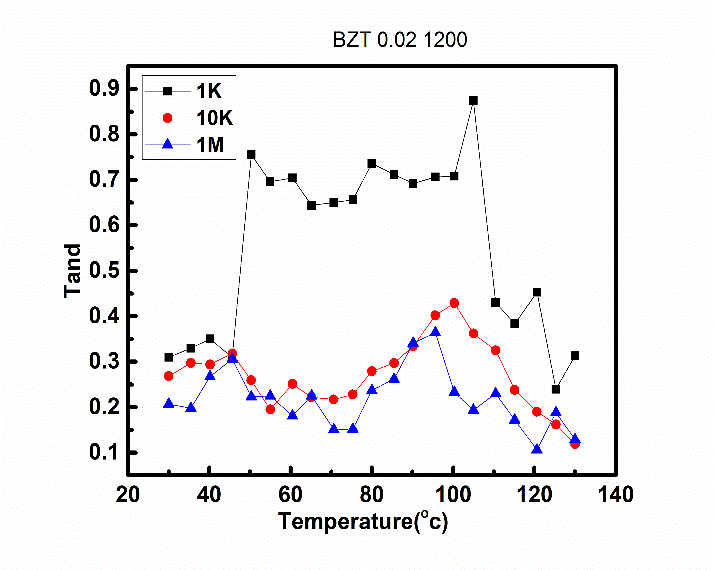

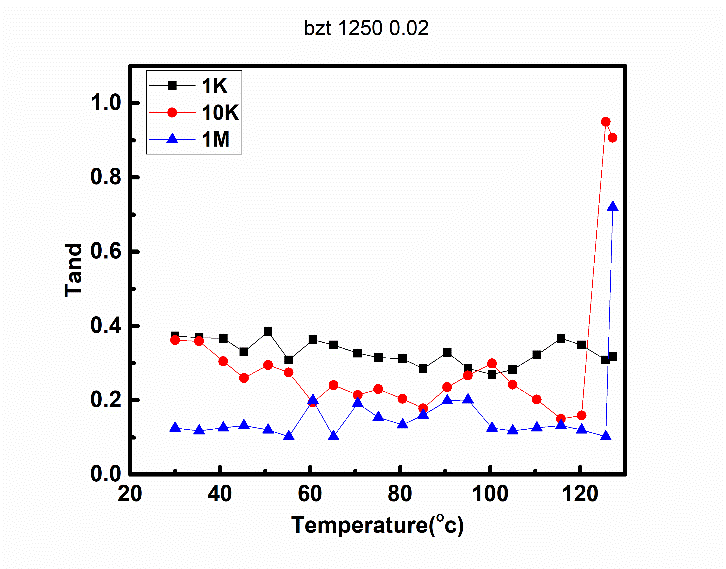


**(a)**

**(d)**

**(c)**

**(b)**

**Figure S3:** (a-b) Temperature dependant dielectric studies of BZT- 1250, BZT-1275, (c-d)

Temperature dependant Loss profile of BZT- 1250, 1275


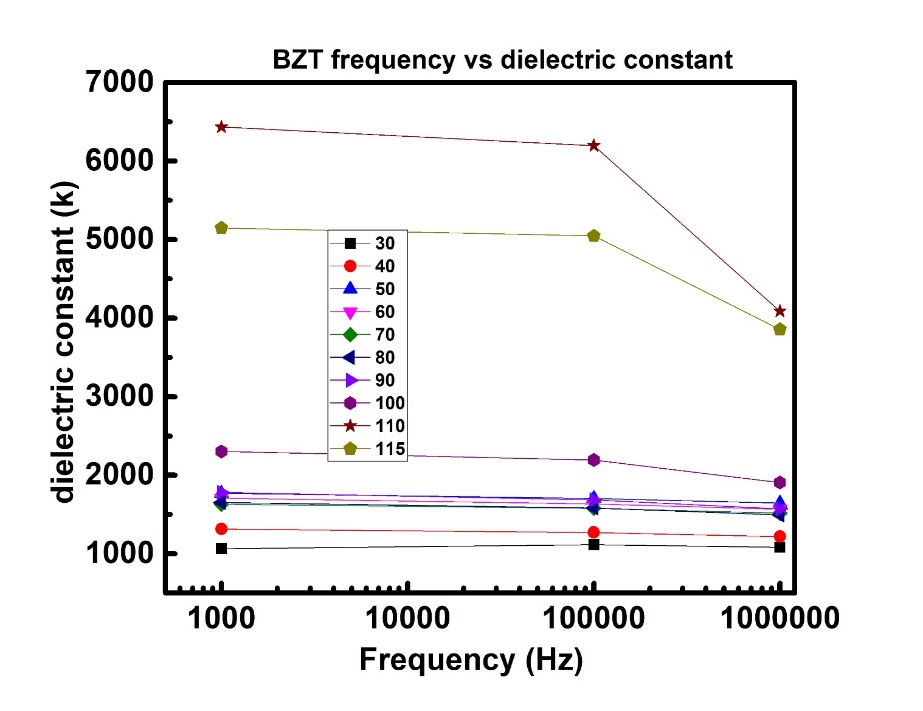

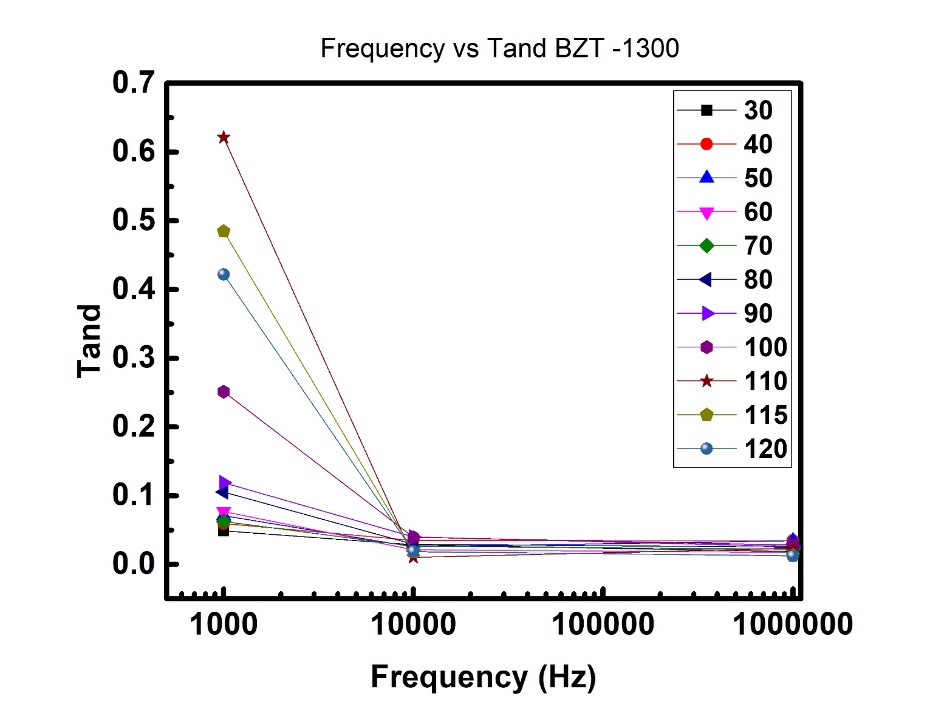


**(a)**

**(b)**

**Figure S4:** (a) Frequency dependant dielectric studies of BZT, (b) Frequency dependant Loss profile of BZT

Fig. S4 (a-b) depicts the frequency-dependent relative permittivity and tand of BZT1300 which are in the range of 1 K to 1M. The dielectric constant and loss of the BZT1300 is observed as inversely proportional to the frequency. The phenomenon is well known. At lower frequencies dipolar polarization domains and as the frequency increases the dipoles cannot follow the oscillations and die out. Because the reason is all types of polarizations (electronic, ionic, orientation and space charge polarization) are active in the low-frequency region. At high frequencies, only electronic polarization is active. The present work BZT also follows the same trend.


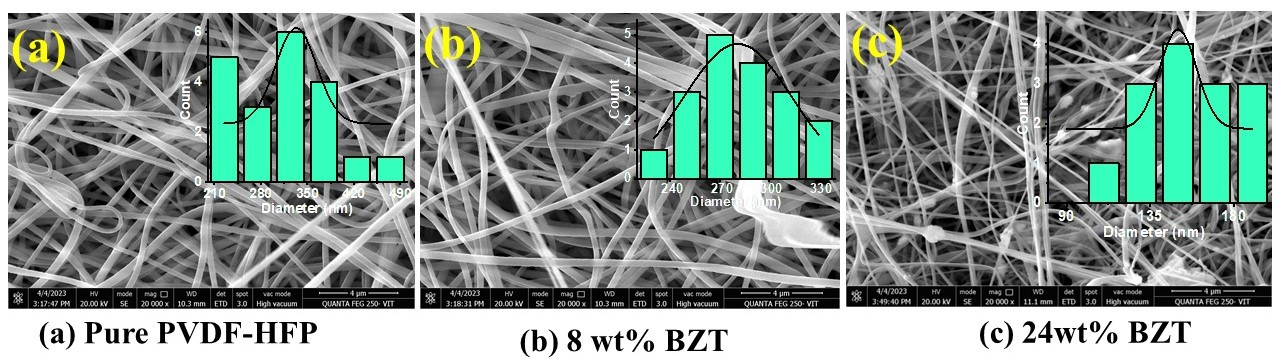


**Figure S5:** (a) SEM images of BZT composite fibers.


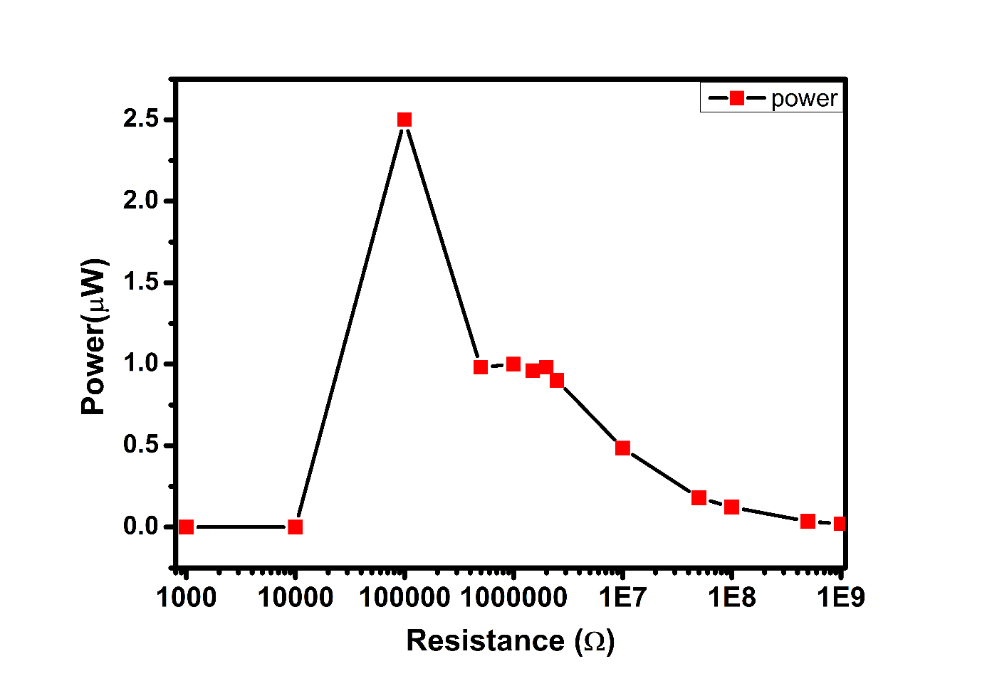


**Figure S6:** The output power of the PENG as a function of the variable loading resistances.


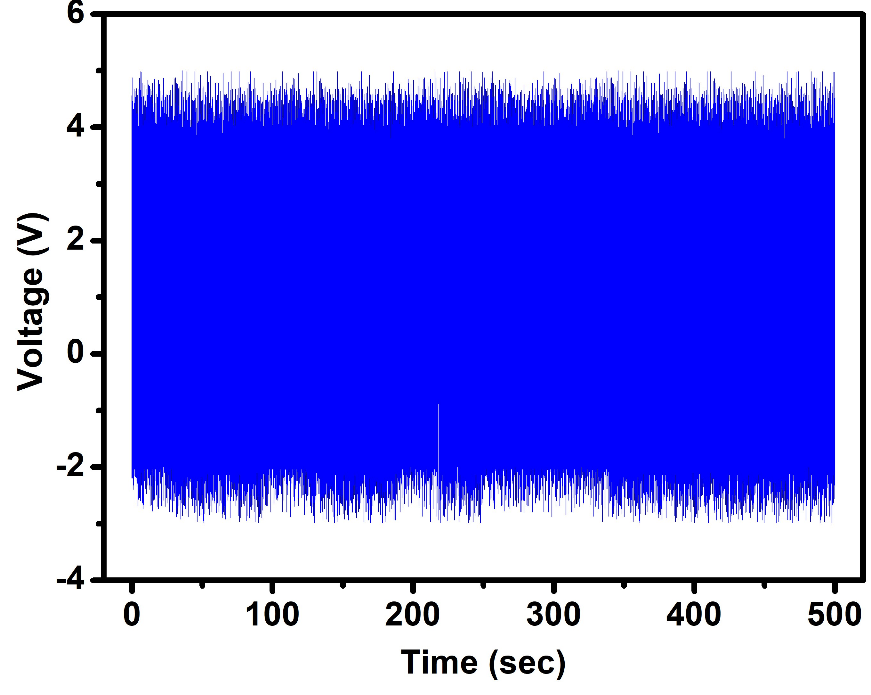


**Figure S7:** Long-term stability test of the PENG


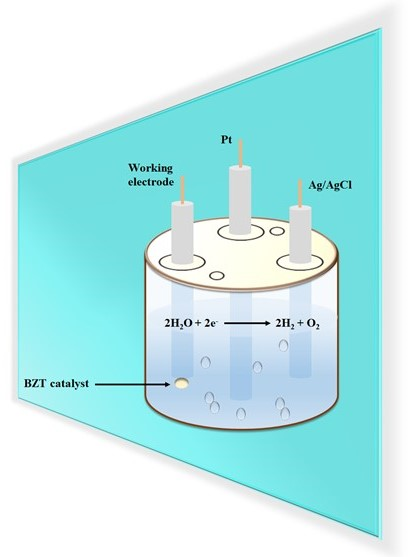


**Figure S8:** schematic of water splitting





**Figure S9:** Stability for BZT with before and after 2000 cv cycles in 0.5M H_2_SO_4_

**Table S1:** Energy storage efficiency varying at different electric fields

| S. No` | Composition@ applied field (KV/cm) | P_max_  (μC/cm^2^) | P_r_  (μC/cm^2^) | E_c_  _(KV/cm)_ | W_r_  (mJ/cm^3^) | W_l_  (mJ/cm^3^) | Efficiency (%) |
| --- | --- | --- | --- | --- | --- | --- | --- |
| 1 | (BZT) @ 2.3 | 1 | 0.01 | 0.5 | 0.74 | 0.84 | 46 |
| 2 | (BZT)@ 6.9 | 6.2 | 2.6 | 1.9 | 7.59 | 15.14 | 33.3 |
| 3 | (BZT) @ 9.3 | 6.9 | 3.1 | 2.1 | 11.5 | 25.27 | 31.2 |
| 4 | (BZT)@ 10.4 | 7.6 | 3.52 | 2.3 | 13.45 | 30.25 | 30.7 |

**Table S2:** Variation of β phase % with different BZT wt%

| Composition | β- Phase (%) |
| --- | --- |
| Raw PVDF-HFP | 55.8 |
| PVDF-HFP | 70.1 |
| BZT (8 W%) | 78.6 |
| BZT (16 W%) | 82.9 |
| BZT (24 W%) | 77.3 |

**Table S3: Comparison of BZT Tafel slope values with literature**

| Electrode | Electrolyte | Overpotential (mV) | Tafel slope  (mV/dec) | References |
| --- | --- | --- | --- | --- |
| CoFe_2_O_4_ nanorods | 1M KOH | 342 | 95 | ^1^ |
| CoNi NSFs + x% Se (x = 0.15) | 0.5M H_2_SO_4_ | 173.5 | 91 | ^2^ |
| Co_0.9_Ni_0.1_Ferrite | 1M KOH | 419 | 81.1 | ^3^ |
| ZnFe_2_O_4_ | 1M KOH | 520 | 144 | ^4^ |
| MgFe_2_O_4_ | 1M KOH | 402 | 241 | ^5^ |
| PrBaCo_2_O_5.5_ | 0.1M KOH | 245 | 89 | ^6^ |
| BSCFP_0.05_ | 0.1M KOH | 337 | 103 | ^7^ |
| SrCo_0.7_Fe_0.25_Mo_0.05_O_3_- d (SCFM_0.05_) | 1M KOH | 323 | 94.21 | ^8^ |
| BZT | 0.5M H_2_SO_4_ | 496 | 77 | This work |
| BZT | 1M KOH | 579 | 151 | This work |

**References:**

1. Ding, Y. *et al.* Single-walled carbon nanotubes wrapped CoFe2O4 nanorods with enriched oxygen vacancies for efficient overall water splitting. *ACS Appl. Energy Mater.* **2**, 1026–1032 (2018).

2. Sadiq Mohamed, M. J. *et al.* Se-Doped Magnetic Co-Ni Spinel Ferrite Nanoparticles as Electrochemical Catalysts for Hydrogen Evolution. *ACS Appl. Nano Mater.* (2023) doi:10.1021/acsanm.3c00464.

3. Chamani, S., Khatamian, M., Peighambardoust, N. S. & Aydemir, U. Microwave-Assisted Auto-Combustion Synthesis of Binary/Ternary Co x Ni1− x Ferrite for Electrochemical Hydrogen and Oxygen Evolution. *ACS omega* **6**, 33024–33032 (2021).

4. Belhadj, H., Messaoudi, Y., Khelladi, M. R. & Azizi, A. A facile synthesis of metal ferrites (MFe2O4, M= Co, Ni, Zn, Cu) as effective electrocatalysts toward electrochemical hydrogen evolution reaction. *Int. J. Hydrogen Energy* **47**, 20129–20137 (2022).

5. Maitra, S., Mitra, R. & Nath, T. K. Investigation of electrochemical performance of sol-gel derived MgFe2O4 nanospheres as aqueous supercapacitor electrode and bi-functional water splitting electrocatalyst in alkaline medium. *Curr. Appl. Phys.* **27**, 73–88 (2021).

6. Sun, Q. *et al.* Double perovskite PrBaCo2O5. 5: An efficient and stable electrocatalyst for hydrogen evolution reaction. *J. Power Sources* **427**, 194–200 (2019).

7. Zhang, Z. *et al.* Enabling efficient hydrogen-evolution reaction over perovskite oxide electrocatalysts through phosphorus promotion. *Int. J. Hydrogen Energy* **45**, 24859–24869 (2020).

8. Zhang, Z., Chen, Y., Dai, Z., Tan, S. & Chen, D. Promoting hydrogen-evolution activity and stability of perovskite oxides via effectively lattice doping of molybdenum. *Electrochim. Acta* **312**, 128–136 (2019).
